# Cancer cells forgo translating mRNA transcribed from genes of nonspecialized tasks

**DOI:** 10.1002/2211-5463.13787

**Published:** 2024-03-11

**Authors:** Mahmoud Ahmed, Trang Minh Pham, Hyun Joon Kim, Deok Ryong Kim

**Affiliations:** ^1^ Department of Biochemistry and Convergence Medical Sciences, Institute of Health Sciences Gyeongsang National University College of Medicine Jinju South Korea; ^2^ Department of Anatomy and Convergence Medical Sciences, Institute of Health Sciences Gyeongsang National University College of Medicine Jinju South Korea

**Keywords:** cancer, gene expression, task specialization, transcription, translation

## Abstract

The coupling of transcription and translation enables prokaryotes to regulate mRNA stability and reduce nonfunctional transcripts. Eukaryotes evolved other means to perform these functions. Here, we quantify the disparity between gene expression and protein levels and attempt to explain its origins. We collected publicly available simultaneous measurements of gene expression, protein level, division rate, and growth inhibition of breast cancer cells under drug perturbation. We used the cell lines as entities with shared origin, different evolutionary trajectories, and cancer hallmarks to define tasks subject to specializing and trading‐off. We observed varying average mRNA and protein correlation across cell lines, and it was consistently higher for the gene products in the cancer hallmarks. The enrichment of hallmark gene products signifies the resources invested in it as a task. Enrichment based on mRNA or protein abundance corresponds to the relative resources dedicated to transcription and translation. The differences in gene‐ and protein‐based enrichment correlated with nominal division rates but not growth inhibition under drug perturbations. Comparing the range of enrichment scores of the hallmarks within each cell signifies the resources dedicated to each. Cells appear to have a wider range of enrichment in protein synthesis relative to gene transcription. The difference and range of enrichment of the hallmark genes and proteins correlated with cell division and inhibition in response to drug treatments. We posit that cancer cells may express the genes coding for seemingly nonspecialized tasks but do not translate them to the corresponding proteins. This trade‐off may cost the cells under normal conditions but confer benefits during stress.

## Graphical abstract

Within eukaryotic cells, DNA is first transformed into mRNA (transcription), followed by the conversion into proteins (translation). Our findings reveal that genes associated with functions that are not actively sustained undergo transcription without subsequent translation into proteins. This pattern incurs an energy cost diverted away from replication and division but offers resilience in challenging conditions.

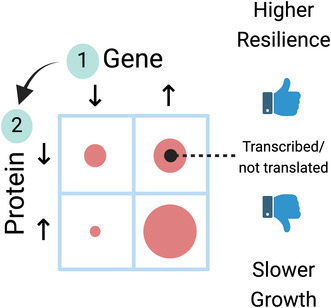

**Abbreviations**
ECDF: empirical cumulative distribution function
FDR: false‐discovery rate
FE: fixed effect
LINCS: library of integrated network‐based cellular signatures
MS: mass spectrometry
NES: normalized enrichment score
PCC: Pearson's correlation coefficients
RPKM: reads per kilobase transcripts per million

Transcription and translation are coupled in prokaryotes [1]. The coupling could, among other benefits, regulate mRNA stability and reduce nonfunctional transcripts [2]. Eukaryotes evolved alternative mechanisms to perform these functions; hence, the two processes are disconnected [3]. The separation of the two processes is spatial and functional, with several layers of regulation in between. We expect the gene expression and protein level measurements to be correlated, but the observed correlation is low [4, 5]. This lack of concordance between the mRNA and protein levels is probably due to these regulatory mechanisms between transcription and translation steps. For example, genes that share common regulators are differentially expressed or code for secretory proteins have a similar concordance and differ in aging and developing cells [6, 7, 8].

Evolutionary theory has been used to explain different aspects of cancer cell behavior. In particular, multi‐tasking and specialization were suggested to influence intra‐tumor heterogeneity and drug response [9, 10]. Task specialization allows for the possibility of trade‐offs. The trade‐off between proliferation and metastasis of cancer cells is the most studied example. Simulations have shown how trade‐offs between tasks could work in the context of cancer. Glioblastoma cells switch from proliferative to invasive phenotype in hypoxic microenvironments rather than transforming to the more aggressive [11]. Godlewski *et al*. [12] suggested a possible mechanism for the phenotype switching in glioma cells. More recent work has made apparent the nonstatic dynamic nature of the tumor growth [13]. In simulated models, glioma cell turnover affects the tumor growth rate and the proliferation/migration trade‐off. Increasing cell turnover slowed the overall growth and accelerated the evolution of proliferation in the interior and migration at the edge of the tumor.

Here, we employ evolutionary theory to explain the discordance between gene expression and protein level measurement. We collected publicly available simultaneous measurements of gene expression, protein level, division rate, and growth inhibition of breast cancer cells under drug perturbation. The cell lines represent entities with shared origins and different evolutionary trajectories. Moreover, the cancer hallmarks define the tasks subject to specializing and trading‐off. We calculated enrichment scores of the hallmarks based on mRNA or protein abundance to estimate relative resources dedicated to transcription and translation (Fig. 1). Similarly, the range of enrichment scores within each cell signifies the resources invested in each task. We observed a pattern of hallmarks mRNA–protein correlation, difference, and range of enrichment of the hallmark genes and proteins that correlated with cell division and inhibition in response to drug treatments. We posit that cancer cells vary their resource investment in gene transcription and translation depending on the degree of specialization. This behavior may cost the cells under normal conditions but confer benefits during stress.

**Fig. 1:**
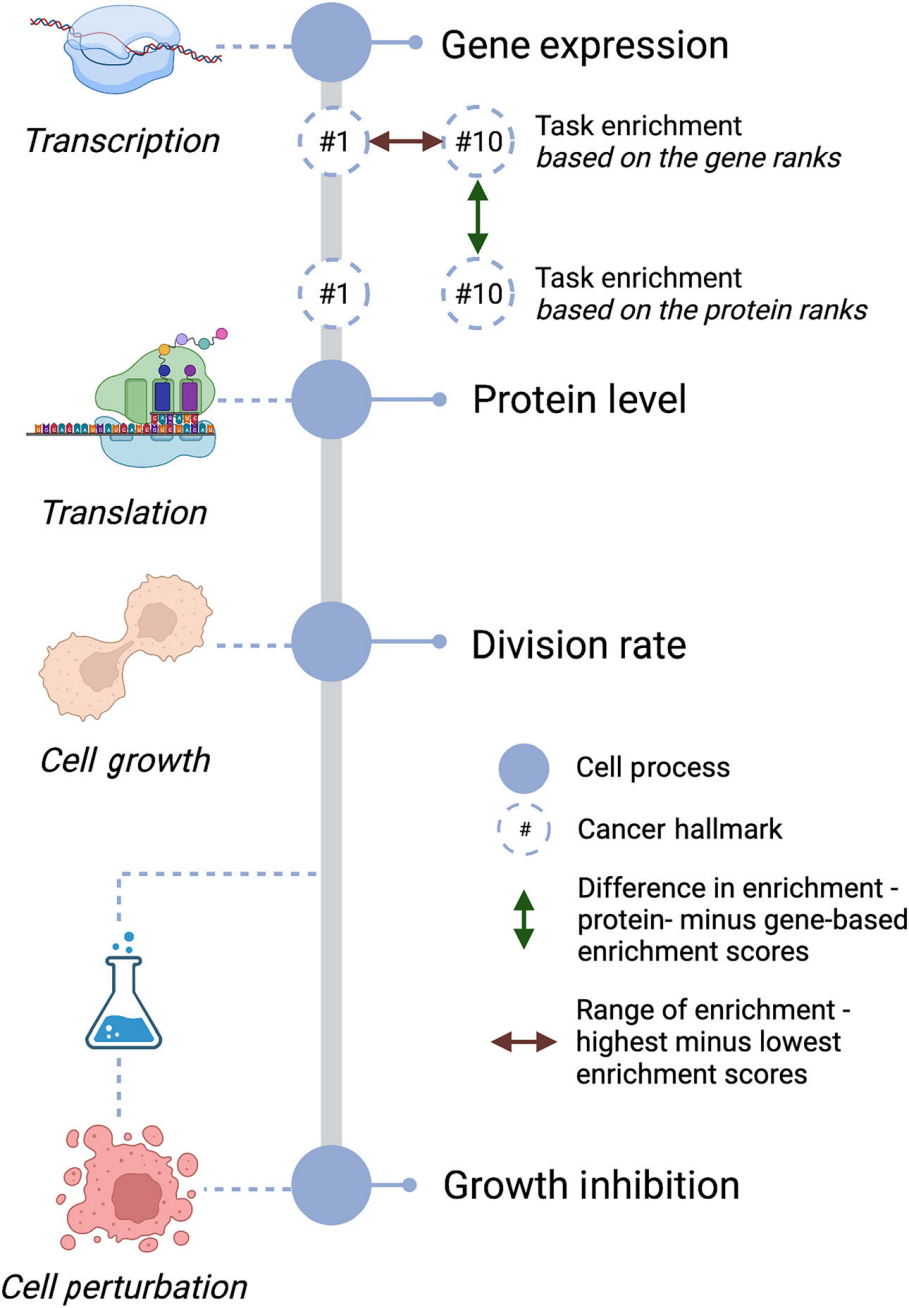
Workflow of the study. Cell growth proceeds by transcribing and translating the required gene products into proteins. We correlated the gene expression and the protein level and studied the association of this correlation with division rates and growth inhibition under drug perturbations (*N* = 101). Moreover, we calculated the enrichment of cancer hallmarks (tasks) based on breast cancer cells' gene and protein levels (*N* = 32). The difference and range of the enrichment scores of the hallmarks were also investigated in relation to the division rate and perturbed growth of some of the breast cancer cells (*n* = 11).

## Materials and methods

### Gene expression of breast cancer cells

We obtained gene expression data (RNA‐seq) of breast cancer cell lines (*N* = 32) from the library of integrated network‐based cellular signatures (LINCS) (Table S1). This dataset was generated and processed as described in Ref. [14]. Briefly, total RNA was extracted for growing breast cancer cell lines. Libraries of mRNA were prepared and assessed for quality. cDNA was synthesized and evaluated for quality. cDNA was sequenced and mapped to the human genome GRCh37. The read counts were normalized to reads per kilobase transcripts per million (RPKM). The processed RPKM was downloaded from http://lincs.hms.harvard.edu/data/HMS_Dataset_20348.zip.

### Protein levels of breast cancer cells

We obtained proteomics data of breast cancer cell lines (*N* = 32) from the LINCS (Table S1). This dataset was generated and processed as described in Ref. [14]. Briefly, cell cultures were precipitated, acidified, and filtered to extract protein aliquots. Peptides were labeled and pooled for high‐performance liquid chromatography separation. Tandem mass tags quantification was performed using mass spectrometry (MS). Raw data were converted into mzXML and corrected for monoisotopic *m/z* measurements and erroneous peptide charge state assignments; then, MS/MS spectra were assigned. The processed protein abundance values were downloaded from http://lincs.hms.harvard.edu/data/HMS_Dataset_20352.zip.

### Division rate and growth perturbations of breast cancer cells

Cell viability measurements under treatments with different drugs (*N* = 101) in the breast cancer cell lines (*n* = 11) were generated by (Table S1) [15] and downloaded from https://lincs.hms.harvard.edu/db/datasets/20268/results?search=output_type=.csv. The data are presented in the form of the maximum growth inhibition values of each drug, which considers the baseline replication rate. These values were plotted against the correlations between the average gene expression profile of the cell lines and their fold change in response to treatments with the same drugs.

### Cancer hallmark gene sets

Lists of genes involved in the 10 cancer hallmarks were compiled previously [16, 17]. Briefly, Zhand *et al*. performed a literature search for terms related to the concepts of each hallmark. The terms were mapped to KEGG pathways (*N* = 301) [18]. Genes involved in the pathways relevant to every hallmark were manually confirmed and curated to the literature and multiple data sources. These included somatic mutations, DNA‐methylation patterns, and copy number variations in cancer patients. We used the list of hallmark genes and proteins (*N* = 2940) to calculate the enrichment in wild‐type (WT) cell lines and in responses to drug treatments. The hallmarks are #1, Activating Invasion and Metastasis (*n* = 1150); #2, Tumor‐Promoting Inflammation (*n* = 649); #3, Inducing Angiogenesis (*n* = 509); #4, Evading Immune Destruction (*n* = 610); #5, Enabling Replicative Immortality (*n* = 309); #6, Sustaining Proliferative Signaling (*n* = 1314); #7, Resisting Cell Death (*n* = 1194); #8, Evading Growth Suppressors (*n* = 710); #9, Genome Instability and Mutation (*n* = 232); #10, Reprogramming Energy Metabolism (*n* = 462). We generated a random gene set (*N* = 500) by randomly sampling from all known genes for testing purposes.

### Gene set enrichment analysis

Gene expression and protein level values were used to make ranked lists of gene products. Enrichment scores were calculated in each list as the over‐representation of the cancer hallmark gene sets at the top or the bottom of the list. Enrichment scores were considered significant when the false‐discovery rate < 0.2. This analysis was applied using the clusterprofiler r package [19].

### Correlation analysis

We used the LINCS profiles to calculate Pearson's correlation coefficients (PCC) between gene expression and protein levels in each cell line and between breast cancer cell lines (*N* = 32). We recalculated the PCC for groups of gene products in the cancer hallmark gene sets. We calculated the PCC between the difference and range of enrichment scores based on the gene or protein ranks with the division rate and the growth inhibition of a subset of breast cancer cell lines (*n* = 11) treated with different drugs (*N* = 101).

### Source code and reproducibility

The analysis was conducted in r (4.0) using bioconductor (3.11) [20, 21]. The docker image and code of this analysis are available on https://hub.docker.com/r/bcmslab/task and https://github.com/BCMSLab/task_separation.

## Results

### Cancer cells radially express specific hallmark gene products

**Fig. 2:**
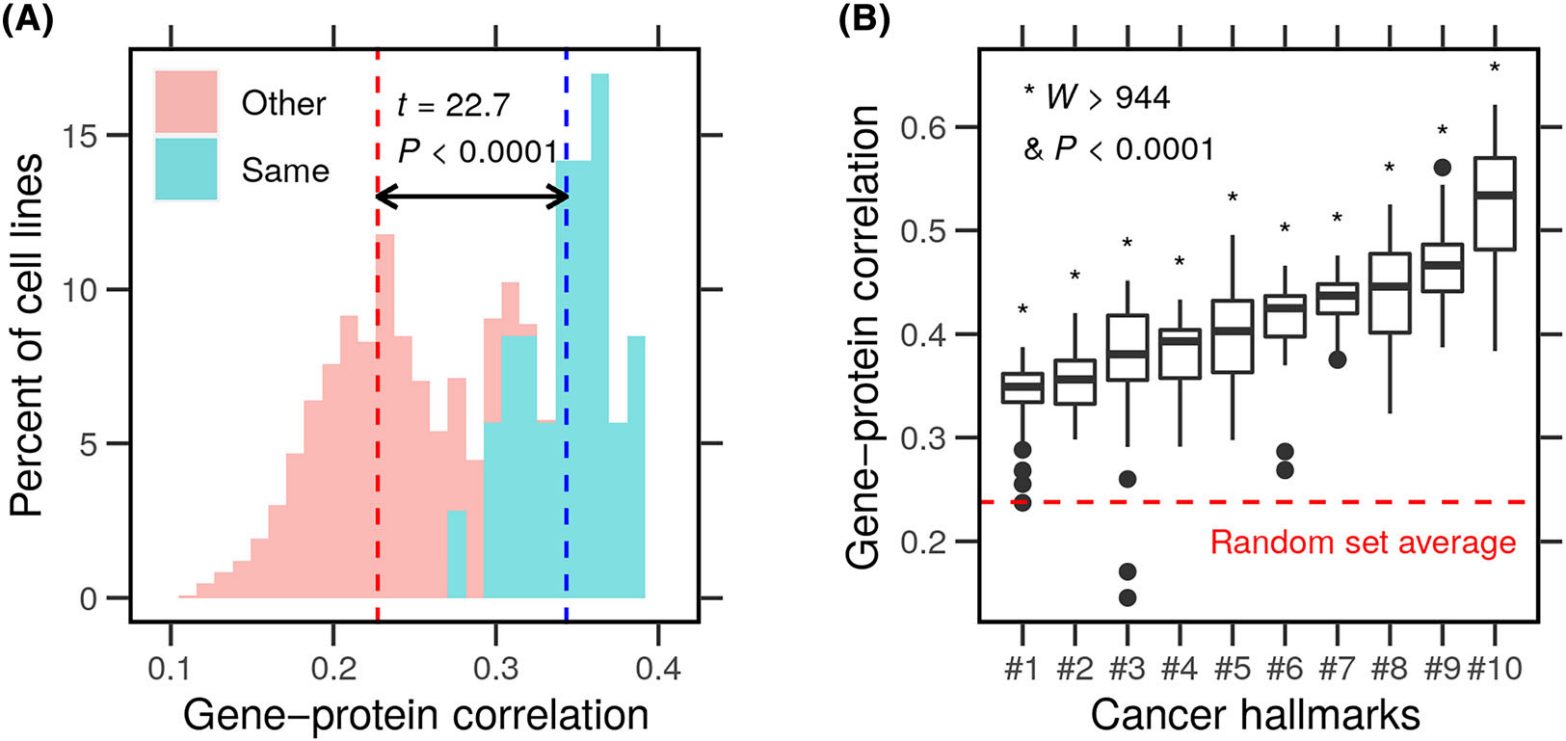
Concordance of cancer hallmark gene expression and protein level. Gene expression and protein level profiles of breast cancer cell lines (*N* = 32) were obtained from the LINCS. (A) A histogram of PCC between gene expression and protein levels in the same (blue) or in other cells (red). (B) Gene products were grouped into 10 cancer hallmark sets, and the correlation (PCC) was recalculated in every cell line and shown as box plots. The hallmarks were ordered by the median correlation, and the same order was followed hereafter. Test statistics of the *t*‐test (*t*) and Wilcoxon test (*W*) are shown. *P < 0.0001.

We used the mRNA and protein abundance in breast cancer cell lines to understand the relationship between transcription and translation. Simple measures of correlation reflect the degree of concordance between the two processes. Pearson's correlation coefficient estimates the normalized covariance of the two variables (mRNA and protein), assuming a linear relationship. The average correlation of mRNA and protein levels in the same cell was higher (*r* = 0.34) compared with in other cells (*r* = 0.23) (Fig. 2A). Moreover, Spearman's rank correlation coefficient assumes a monotonic relationship and uses the ranks rather than the absolute value of the variables. The correlation of the gene and protein ranks yielded similar values (ρ = 0.34 and 0.23). The difference between these averages was significant (*t* = 22.71, df = 35.33, *P* < 2.2 × 10^−16^) in a two‐sample *t*‐test. Furthermore, the empirical cumulative distribution function curves (ECDF) of the correlations in the same cell and other cells were drawn from different distributions in the Kolmogorov–Smirnov test (*D* ^+^ = 0.92, *P* < 2.2 × 10^−16^) (Fig. S1A). This shows a moderate correlation between the genes's mRNA and the corresponding protein. Next, we asked whether the mRNA–protein correlation was similar for gene products involved in cancer hallmarks.

We recalculated the correlation of mRNA and proteins for 10 sets of gene products corresponding to cancer hallmarks. We found that the genes involved in one or more cancer hallmarks have consistently higher mRNA–protein correlation in most cells (Fig. 2B). The average correlation in the hallmarks was higher than the one found in a set of random genes (*N* = 500; sampled from all known genes) (*W* > 944, df = 10, *P* < 5.46 × 10^−19^). Furthermore, we regressed the mRNA–protein correlations on the hallmarks and the random set. ANOVA test (*F* = 80, df = 10, *P* < 2.2 × 10^−16^) showed a consistently higher mean correlation compared with the random set sampled from all known genes (Fig. S1B). We turned next to exploring the cell resource investment in gene transcription and protein synthesis.

### Gene products in specific cancer hallmarks are translated into proteins

Enrichment of a gene set signifies the amount of resources dedicated to expressing its gene products (mRNA and proteins). In addition, the difference between the mRNA‐based enrichment and the protein‐based could signify in relative terms the resources invested in transcription and protein synthesis. Similarly, comparing the most to the least enrichment scores of the gene sets indicates the degree to which the cell allocates resources to specific gene sets. Here, gene products were ranked based on the mRNA or the protein levels. Second, we obtained enrichment scores for the lists of genes corresponding to the cancer hallmarks. Enrichment scores were further normalized by the mean positive and negative scores separately. We computed the difference between the mRNA‐based and the protein‐based scores for each gene set and the range of enrichment as the difference between the highest and lowest enrichment scores in each ranked list. We also correlated these measures with nominal division rates and growth inhibition under treatment with different drugs.

**Fig. 3:**
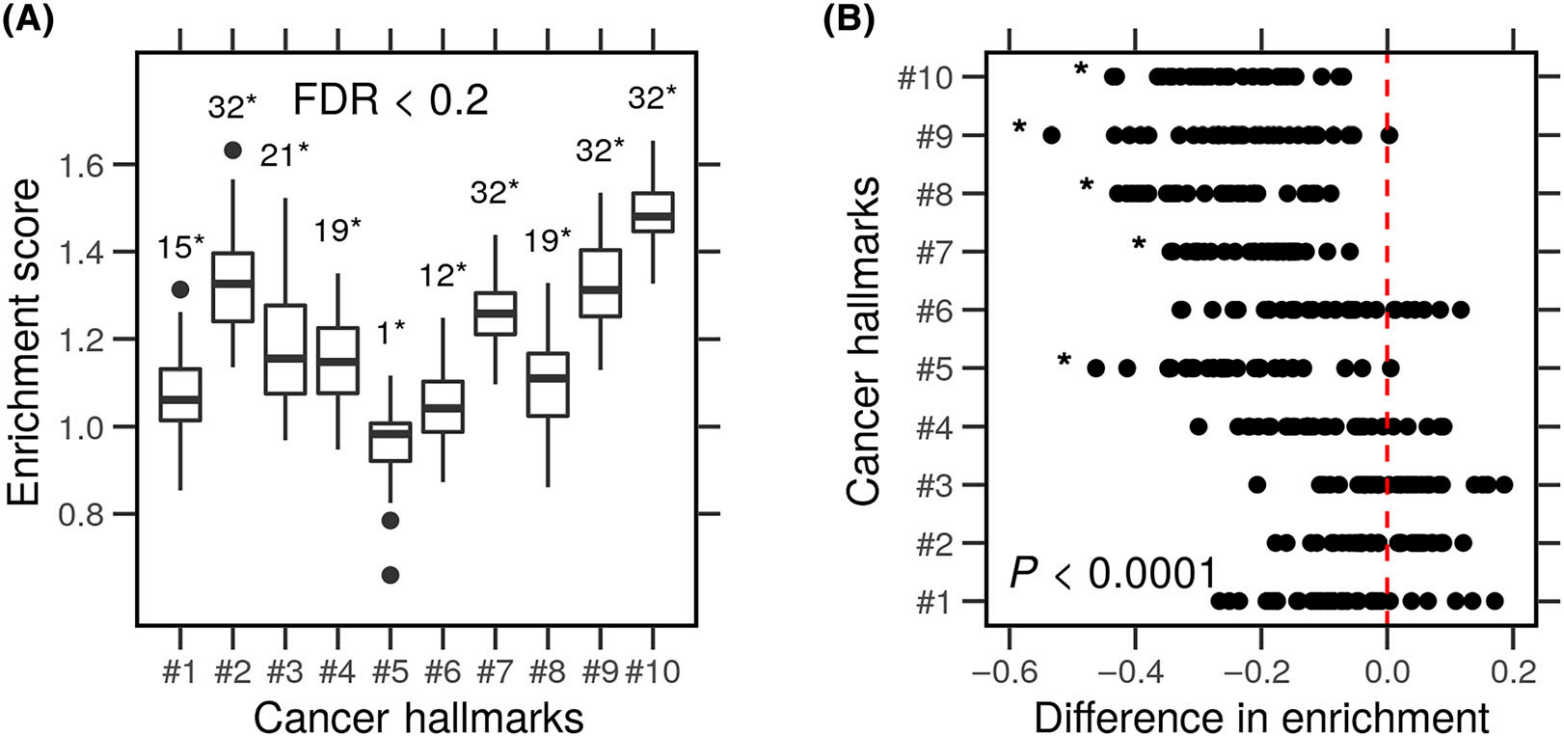
Correlation between the gene‐protein concordance and cancer hallmarks enrichment. Gene expression and protein level profiles of breast cancer cell lines (*N* = 32) were obtained from the LINCS. Genes products were ranked based on the gene expression or protein level, and the over‐representation of the hallmark gene sets was calculated for each cell line. (A) Box plots of the enrichment scores based on the gene expression of each cell. *FDR < 0.2. (B) The difference in enrichment scores based on genes or protein levels of cancer hallmarks in every cell line. *P < 0.0001.

As expected, most hallmarks were enriched in the mRNA ranked list (Fig. 3A). The lowest normalized enrichment score was always positive (NES > 0.6). Most cells had at least one hallmark significantly enriched at an appropriate false‐discovery rate (FDR < 0.2) (Fig. S2). The genes coding for hallmark function are expected to be actively transcribed in cancer cells, hence the positive enrichment scores. Four hallmarks, ‘Tumor‐Promoting Inflammation (#2), Resisting Cell Death (#8), Genome Instability and Mutation (#9), and Reprogramming Energy Metabolism (#10)’, were significantly enriched in all cell lines (*N* = 32). Moreover, cell types vary with regard to the hallmarks of which their gene products are most actively transcribed.

Subtracting the mRNA‐based enrichment scores from the protein‐based scores consistently yielded significant differences across the cell types (Fig. 3B). Certain hallmark gene products are more enriched at the mRNA level, compared with the protein level. We used bootstrapping to test the robustness of these differences. First, we obtained random samples (*N* = 32) from the mRNA‐ and protein‐based scores and calculated the difference between them. The procedure was repeated multiple times (*N* = 10 000). The absolute difference in the random samples was compared with the absolute difference in each gene set to calculate a *P*‐value. Five hallmark gene sets tested significant (MD = −0.17, *P* < 0.0001). This difference in enrichment scores indicates selective investment of resources in transcription and translation of the hallmark gene products in each cell. We explore next how the cell behavior correlates with cell division.

### Separating transcription and translation impacts the cell division

**Fig. 4:**
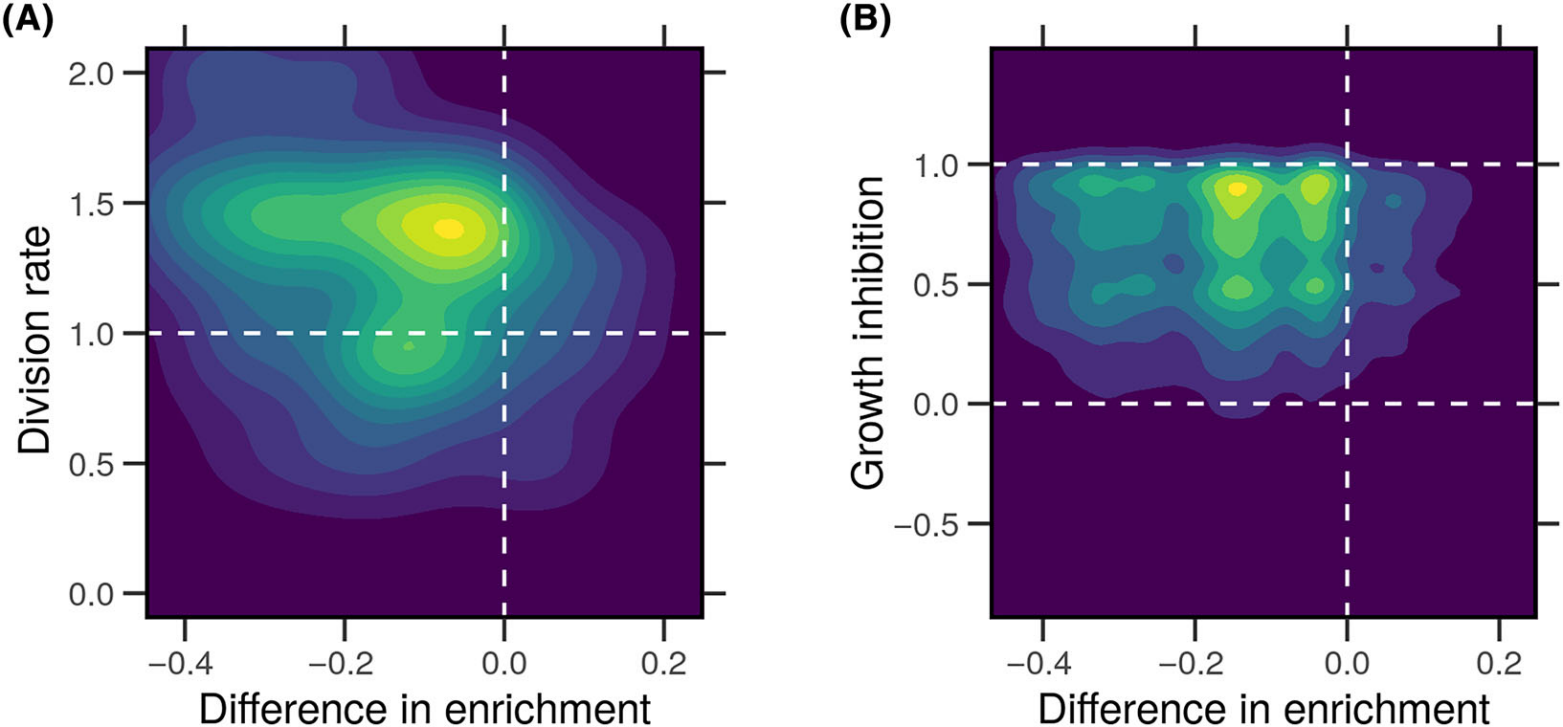
Cancer hallmarks enrichment differences and cell growth. Gene expression and protein level profiles of breast cancer cell lines (*n* = 11) were obtained from the LINCS. Genes products were ranked based on the gene expression or protein level, and the over‐representation of the hallmark gene sets was calculated for each cell line. The difference in enrichment scores based on genes or protein levels of cancer hallmarks in every cell line. Cell viability data of the same breast cancer cell lines (*n* = 11) without or with drug perturbations (*N* = 101) were obtained from LINCS. (A) The density distribution of the differences in enrichment scores and the cell nominal division rates. (B) The density distribution of the differences in enrichment scores and the cell growth inhibition values under drug treatments.

To further explore the significance of the differences in enrichment scores of hallmarks, we plotted them against the division rate and the growth inhibition in response to drug perturbation in a subset of the breast cancer cell lines. In most instances, the difference in enrichment is associated with the division rates of the WT nontreated cells (Fig. 4A). The overall correlation between the two variables was positive and significant (PCC = 0.32, *P* = 0.00006). As we expect, most cells are able to balance investment in transcription and translation, achieving moderate growth. However, cells with more minor discrepancies in enrichment grow the slowest, and cells with the highest difference in enrichment (investing in transcription and selectively regulating translation) consistently have high growth. To contrast this with stressful conditions, we utilized data from a subset of the cell lines under treatment with different compounds.

### Cancer cells are robust to differences in the transcription–translation

**Fig. 5:**
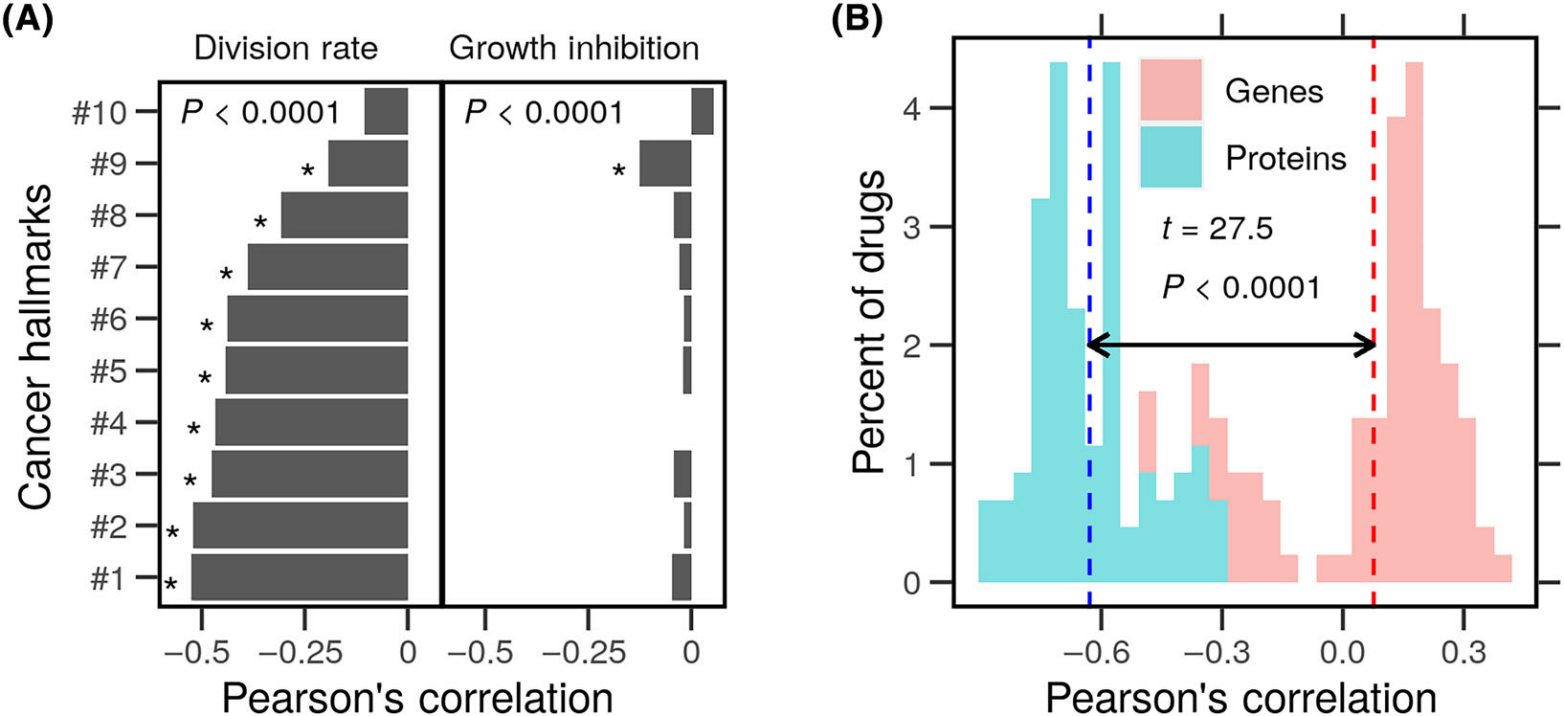
Difference and range of enrichment of the cancer hallmarks and their correlation to cell growth. Gene expression and protein level profiles of breast cancer cell lines (*n* = 11) were obtained from the LINCS. Genes products were ranked based on the gene expression or protein level, and the over‐representation of the hallmark gene sets was calculated for each cell line. Cell viability data of the same breast cancer cell lines (*n* = 11) without or with drug perturbations (*N* = 101) were obtained from LINCS. (A) The PCC between the differences in enrichment scores of cancer hallmarks and the cell nominal division rates and the cell growth inhibition values under drug treatments are shown as bars. (B) A histogram of the PCC between the range of cancer hallmarks enrichments based on the protein ranks and the nominal division rate of every cell line. *P < 0.0001.

The difference in enrichment scores was not associated with growth inhibition under drug perturbations (PCC = 0.012, *P* = 0.2) (Fig. 4B). Interestingly, cell lines with negative differences in enrichment mRNA and protein‐based scores consistently divided typically or grew always under different treatments. Cells are robust to perturbations in that allocating resources to translating necessary proteins does not incur an extra cost in terms of growth potential. Another way of examining the relationship between growth rates and the differences in enrichment scores based on mRNA or protein ranks is to investigate their correlation coefficients. Interestingly, the correlation between the difference in the enrichment of various hallmarks differed significantly but was generally higher with division rates (PCC = 0.1–0.5, *P* < 0.0001) under normal conditions (Fig. 5A). The fixed effect (FE) of the difference in enrichment on the division rates was always negative (FE = −1.88, *P* < 0.0001). We estimated the effect in the individual hallmarks using separate linear models (Fig. S3A). The estimates of the impact of the difference in enrichment on the division rate were consistently negative and significant. By contrast, there seems to be no correlation (PCC < 0.04, *P* > 0.07) between the difference in enrichment and the response to drug treatments in terms of growth inhibition. Only the ‘Genome Instability and Mutation (#9)’ hallmark had a small but significant correlation with growth inhibition.

The range of enrichment (the difference between the highest and the lowest enriched hallmarks) strongly correlated with the division rate of nontreated cells (Fig. 5B). The more extensive the range of enrichment based on protein ranks, the smaller the division rate. A small but significant correlation was also observed for the range of enrichment based on the mRNA ranks. The ECDF curve of the range of enrichment in the protein ranks lies above (*D* ^+^ = 2.4, *P* < 0.001), and that of the enrichment based on gene ranks lies above a random variable (*D* ^−^ = 1.7, *P* < 0.001) (Fig. S3B). This indicates a broader allocation of resources to transcribe mRNA of the gene products and more selectivity in the translation step.

## Discussion

**Fig. 6:**
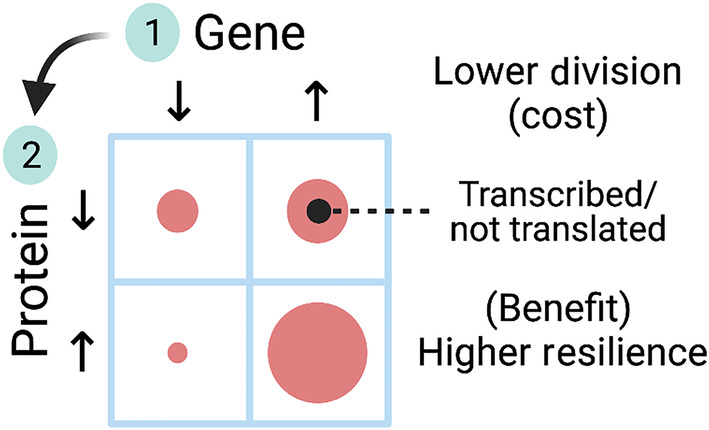
Model of task‐specialized transcription–translation decoupling. Transcription and translation are decoupled in eukaryotes. The transcription of genes and their translation into proteins are required to perform cellular functions. In cancer cells, genes involved in functions that are not actively maintained are still transcribed but not translated into proteins. This arrangement comes at the cost of energy not directed toward replication and division and with the benefit of being resilient in stressful conditions.

Gene expression and protein levels are imperfect measurements of the transcription and translation processes. In eukaryotes, these processes are decoupled. The correlation between gene and protein levels is moderately positive (Fig. 2). We initially predicted that the enrichment of cancer hallmarks would be associated with a stronger correlation between the expression of the genes and the level of proteins involved in each ta due to task specialization. We found that this association holds for some but not other hallmarks (Figs 2B and 3A). The genes of nonspecialized tasks might still be transcribed at the cost of reduced cell growth in normal conditions (Figs 4 and 5A). However, cells could translate these genes into the proteins required to resist stressful conditions (Fig. 5B). A graphic summary of these findings is presented in Fig. 6.

The difference in the enrichment of the genes and the proteins in the cancer hallmarks correlated with the division rate of cancer cells under normal conditions but not their response to drugs (Figs 4 and 5A). This is to say, the transcription of genes involved in nonspecialized cancer tasks consumes cell resources that would otherwise be directed toward replication and division. This difference does not impact the growth rate under stress since no correlation was established to it. The range of enrichment of genes and proteins in the hallmarks correlated with the cell division rate (Fig. 5A). Higher growth was associated with a broader range of gene enrichment and a tight range of protein enrichment. Cells that maintain a tight range of translated proteins exhibit more resistance to drug perturbations.

Cancer cells growing under drug perturbations exhibit different behaviors. The ability of cancer cells to maintain growth under stress depended on the enrichment of proteins involved in the cancer hallmarks, but not the mRNA that might be already present (Fig. 5B). The higher the enrichment of both genes and proteins involved in a task, the lower (on average) the growth inhibition in response to most drugs. However, the relatively lower gene set enrichment was no obstacle to this resistance. These genes are probably transcribed at a moderate level, and cancer cells could induce their translation as needed. This makes the cancer cells resilient in the face of adverse conditions. The current analysis doesn't take into account the differential importance of a particular hallmark to any given cell line. Rather, we identify a pattern of optimization to resources dedicated to the transcription and translation rates of cells depending on the degree of specialization in each cell type.

Gene expression might be regulated separately at the abundance and the activity level (dimmer switch gene regulation) [22]. This represents a possible fine‐tuning mechanism by which genes coding for the nonspecialized tasks are transcribed but not necessarily translated into protein. Moreover, evidence has shown that genes coding for a particular cellular function share post‐transcriptional regulators, which would take to work under stressors such as drug perturbations [8]. The shared regulators may explain the lower disparity between the mRNA and protein levels involved in specific pathways.

Aktipis *et al*. [23] applied life‐history theory to the evolution of cancer. In their perspective, the pace of life histories depends on the availability of resources and the stability of the microenvironment. The faster or slower pace is associated with different cancer hallmarks. In other words, growing tumors are constrained by limited resources. As a result, individual cells compete with each other and normal cells. Therefore, cells direct resources toward specific tasks and trade off others to gain an advantage under selective pressure. Experimental evidence of this phenomenon is well studied in the case of the trade‐off between proliferation and survival. One study showed that breast cancer cells propagate and proliferate less when the need to detoxify oxygen species [24]. In addition, estrogen‐positive and negative cells trade off serine and glutamine metabolism.

Individuals in Pareto efficient systems cannot be made better off without making others worse off. This concept was used to infer tumor tasks and trade‐offs between phenotypes in evolving organisms [25]. To operationalize this procedure, the authors used the weighted average of the inferred archetypes to identify generalist and specialist individuals in the population. They also applied this method to gene expression data from cancer cells to infer the biological tasks [26]. In another report, Hausser *et al*. [10] studied task specialization and trade‐offs in tumors using a similar procedure. The study provided evidence of the enrichment of functions related to a specific task in some tumors (specialists) but not others (generalists). We further tested one of their predictions using an independent dataset and approach. We used gene expression profiles of cancer cells to show that cells are susceptible to drugs that disrupt the tasks in which cells specialize [27].

Cell lines are the end product of separate life histories of evolution, specialization, and trade‐offs. We leverage this property to study cancer cell behavior despite not having access to the process that generates it. The current work does not explicitly provide evidence for the notion of specialization and trade‐offs between cancer tasks. Instead, we use the theory to explain the cell‐selective investment in gene and protein synthesis under different conditions. In addition, we use the cancer hallmarks as the tasks subject to specialization and trade‐offs. This could bias the identification of tasks toward known cancer cell functions rather than identifying them *de‐novo*.

Furthermore, we evaluate the cell investment in each task in relative terms as the enrichment of its gene and protein members. There is no agreement on how to infer tasks from functional data. For example, Plutynski points out that an analysis of life history and trade‐offs should draw on phenotypes and genotypes [28]. We chose the 10 cancer hallmarks to signify tasks important to the survival and growth of cancer cells. The current analysis treats the different tasks as virtually equal in terms of their significance. However, this is not necessarily the case. We could only show that optimizing for one or more tasks could impact cell growth. Future work should emphasize individual tasks and show whether essential functions are differentially impacted by transcription and translation rate differences.

We could not relate the current findings to specific cancer types. This may be attributed to the small number of cell lines in each subtype. Similarly, the cell cycle state would affect the transcription and translation rates. To consider this influence of cell state, we would need a dataset where the cell cycle state is known or a sufficiently big dataset to capture the variation between the cell cycle states. Unfortunately, these conditions are not met with the current dataset and further investigation is needed to differentiate the impact of the cell cycle state and optimization of transcription and translation rates.

## Conflict of interest

The authors declare no conflict of interest.

### Peer review

The peer review history for this article is available at https://www.webofscience.com/api/gateway/wos/peer‐review/10.1002/2211‐5463.13787.

## Author contributions

MA conceived, performed, and reported the analysis and prepared the manuscript. TMP performed the analysis and writing. HJK acquired the funding and edited the manuscript. DRK acquired the funding, supervised, and contributed to the concepts and the writing.

## Supporting information


**Fig. S1.** Testing the concordance of cancer hallmarks, gene expression and protein levels.
**Fig. S2.** Enrichment of the cancer hallmark gene sets in cancer cells.
**Fig. S3.** Correlating the difference and range of enrichment of the cancer hallmarks with cell division.
**Table S1.** Cell lines and available data.

## Data Availability

The data analyzed in this study were obtained from online repositories: https://lincs.hms.harvard.edu/db/datasets/20348, https://lincs.hms.harvard.edu/db/datasets/20352, and https://lincs.hms.harvard.edu/db/datasets/20268.
