# Supplementary material for: Cancer cells forgo translating mRNA transcribed from genes of nonspecialized tasks
Source: FEBS Open Bio. 2024 Mar 11;14(5):793–802. doi: 10.1002/2211-5463.13787 (PMC11073504; doi:10.1002/2211-5463.13787)
Supplement: Supplementary file 1 — Fig. S1. Testing the concordance of cancer hallmarks, gene expression and protein levels. Fig. S2. Enrichment of the cancer hallmark gene sets in cancer cells. Fig. S3. Correlating the difference and range of enrichment of the cancer hallmarks with cell division. Table S1. Cell lines and available data. [file FEB4-14-793-s001.pdf]

Table S1: Cell lines and available data.

Clinical Subtype	Transcriptional Subtype	Cell Line	Growth Rate	Gene Expression	Protein Level
HER2 amplified	Basal A	HCC1954	√	√	√
	Luminal	HCC1419	√	√	√
	Luminal	MDAMB361		√	√
	Luminal	SKBR3	√	√	√
HR positive	Luminal	CAMA1		√	√
	Luminal	HCC1428	√	√	√
	Luminal	MCF7	√	√	√
	Luminal	T47D	√	√	√
TNBC	Basal A	HCC1143	√	√	√
	Basal A	HCC70	√	√	√
	Basal A	MDAMB468		√	√
	Basal A	BT20		√	√
	Basal A	HCC1806	√	√	√
	Basal A	HCC1937	√	√	√
	Basal B	BT549		√	√
	Basal B	HCC1395		√	√
	Basal B	HCC38	√	√	√
	Basal B	MDAMB157		√	√
	Basal B	MDAMB231		√	√
	Basal B	MDAMB436		√	√
	Luminal	MDAMB453		√	√
	Luminal B	CAL120		√	√
	In situ	SUM159			√
	In situ	SUM149			√
	Metastatic	SUM1315		√	√
	Metastatic	CAL51		√	√
	Metastasis	PDX1258		√	√
FGFR altered	Metastatic	MDAMB134		√	√
Unknown	Medullary	PDXHCI002		√	√
	Ductal	HCC1500		√	√
	Fibrocystic	MCF10A		√	√
		PDX1328		√	√

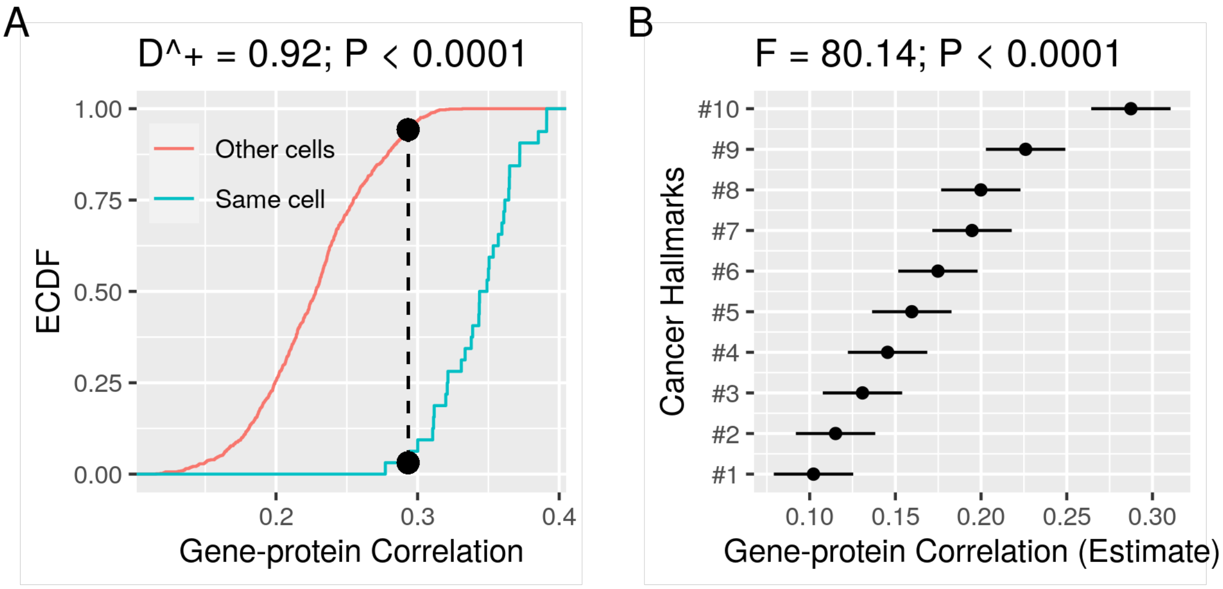

Figure S1: Testing the concordance of cancer hallmarks gene expression, and protein levels. Gene expression and protein level profiles of breast cancer cell lines (n = 32) were obtained from the library of integrated cellular signatures (LINCS). A) The empirical cumulative distribution function (ECDF) of the gene-protein correlation within (Same cell) vs. between cancer cells (Other cells). Kolmogorov–Smirnov (KS) test was applied to test the significance of the shift distance between the curves. B) Coefficients estimates of regressing the gene expression on the protein levels in each of the ten cancer hallmarks. Analysis of variance (ANOVA) was applied to calculate F-statistics and the significance of the overall trend.

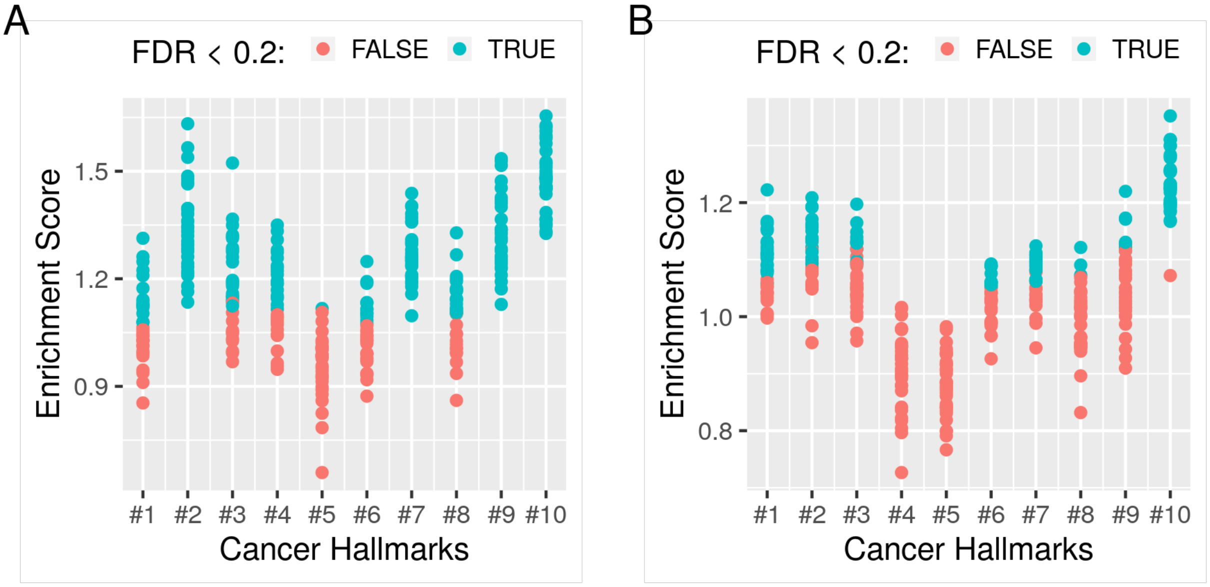

Figure S2: Enrichment of the cancer hallmarks gene sets in cancer cells.
Gene expression and protein level profiles of breast cancer cell lines (n = 32) were obtained from the library of integrated cellular signatures (LINCS). Genes products were ranked based on the A) gene expression or B) protein level, and the over-representation of the hallmarks gene sets was calculated for each cell line. False discovery rate (FDR) was calculated, and enrichment was considered significant if FDR $<$ .2 (blue) otherwise (red).

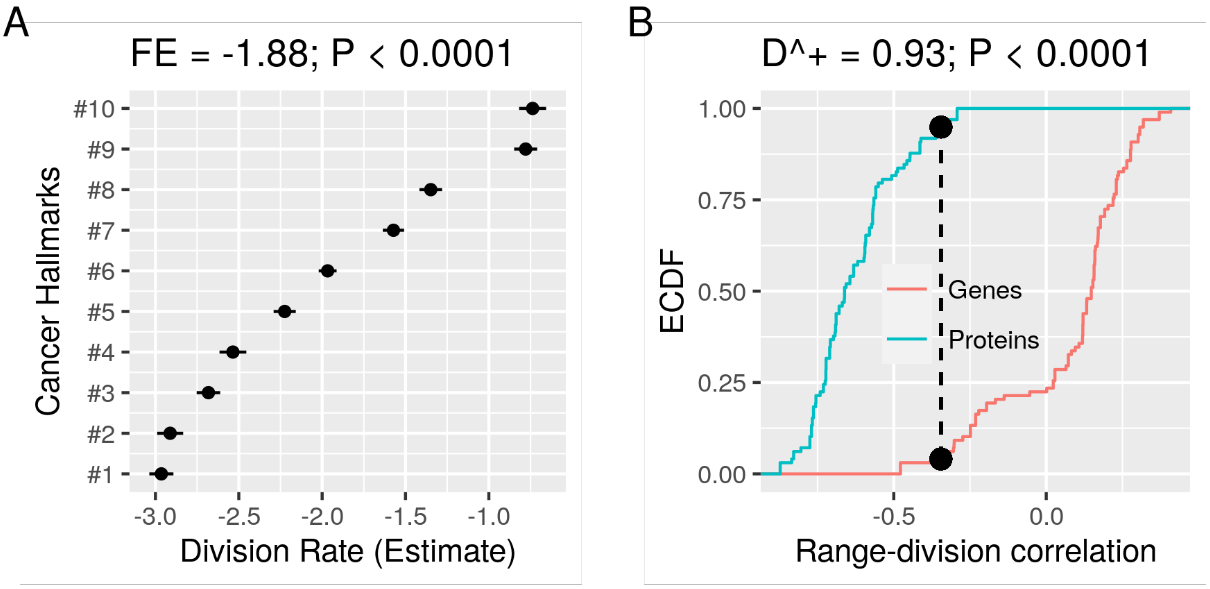

Figure S3: Correlating the difference and range of enrichment of the cancer hallmarks with cell division. Gene expression and protein level profiles of breast cancer cell lines (n = 11) were obtained from the library of integrated cellular signatures (LINCS). Genes products were ranked based on the gene expression or protein level, and the over-representation of the hallmarks gene sets was calculated for each cell line. The difference (protein-based - gene-based) and range (highest - lowest) of enrichment scores of cancer hallmarks in every cell line were calculated. Cell viability data of the same breast cancer cell lines (n = 11) without or with drug perturbations (n = 101) were obtained from LINCS. A) Division rates were regressed on the difference in enrichment in a fixed-effect (FE) model, and the effect in each cancer hallmark was calculated. B) The empirical cumulative distribution function (ECDF) of the range of gene-based (red) and protein-based (blue) enrichment. Kolmogorov–Smirnov (KS) test was applied to test the significance of the shift distance between the curves.
